# Applying discrete choice modelling in a priority setting: an investigation of public preferences for primary care models

**DOI:** 10.1007/s10198-013-0542-8

**Published:** 2013-11-15

**Authors:** Chiara Seghieri, Alessandro Mengoni, Sabina Nuti

**Affiliations:** 1 Laboratorio Management e Sanità, Istituto di Management, Scuola Superiore Sant’Anna, Piazza Martiri della Libertà, 24, 56127 Pisa, Italy; 2 Azienda Sanitaria Unica Regionale, Via Caduti del Lavoro, 40, 60131 Ancona, Italy

**Keywords:** Discrete choice experiment, Primary care organisation, Diagnostic facility, Priority setting, Patient satisfaction and experience survey, Italy, I1

## Abstract

**Objectives:**

The shift toward more innovative and sustainable primary care models in Italy leads policy makers and clinicians to face difficult decisions between options that are all regarded as potentially beneficial. In this study, patient preferences for different primary care models in the Tuscany region of Italy were elicited. The relative importance of different attributes to the surveyed respondents was then examined, as well as the rate at which individuals trade between attributes and the relative value of different service configurations.

**Methods:**

A discrete choice experiment survey explored the following attributes in a stratified random sample of 6,970 adults: primary care provider, diagnostic facilities and waiting time for the visit.

**Results:**

Respondents (3,263) were likely to prefer a consultation by their own general practitioner (GP) and a practice with many diagnostic facilities. The predicted utilities of different service configurations have shown that a “primary care centre” with many diagnostic facilities was preferable to a “solo GP” model or a “group general practice”.

**Conclusions:**

The study demonstrated how a patient choice model could be used by decision makers for developing successful policies that takes into account different healthcare needs, balancing responsiveness with care continuity, equity and appropriateness. Considering that a primary care centre would perform better than a “solo GP”, especially for younger respondents and for those with minor healthcare needs, for a more rapid diffusion of this model policymakers and managers could direct the care of primary care centres towards these targeted subgroups, at least in the first phase.

## Introduction and background

Over the past two decades, healthcare reforms in Western Europe have changed primary care systems, reshaping in particular the organisational role of general practitioners (GPs) and their clinical and managerial activities [1]. A major thesis shared by many countries is the promotion of cooperation among GPs as well as the improvement of inter-professional collaborative team works as a means to spread knowledge, facilitate accountability and, ultimately, improve patient care with limited resources [2].

For some years now, also in Italy primary care organisational models have been frequently reconsidered in order to enhance accessibility and improve coordination, continuity, and comprehensiveness of care, to increase the capacity for efficient, effective and appropriate care, and to provide opportunities for nursing and other healthcare providers to engage in collaborative practice with GPs. Nevertheless, these changes have consistently been supply-led rather than demand-led and the idea that the redefinition of primary care models should be primarily consistent with population needs and preferences is strengthening [3].

Various types of primary care models are currently active in Italy. Traditionally, GPs in Italy have worked in solo practices without any auxiliary staff or institutional links to other GPs.

Over the past 10 years, many local health authorities (LHAs) have tried to reshape the traditional model of primary care by encouraging GPs to participate in collaborative arrangements such as group practices in which GPs share practice space and other resources [4]. The main idea behind such initiatives was the improvement of care continuity by reinforcing service coordination and information-sharing among GPs in a practice. However, apart from these expedients, and patient loyalty to their physician, there were no formal mechanisms to guarantee longitudinal and vertical continuity of care [5]. Moreover, associated GPs did not appear to perform better in terms of meeting LHA pharmaceutical budgets because of the connections formed as a consequence of GP networks [4].

More recently, Italian primary healthcare reform has moved towards a more comprehensive and team-based approach to address population-specific needs and to treat chronic diseases more proactively. In this setting—currently in the experimental phase—professionals from various disciplines (GPs, specialists, out-of-hours doctors, nurses, physiotherapists, psychologists, social workers) provide a broad range of medical and community services covering diagnostic, curative and palliative care, disease prevention, rehabilitation, home care and patient education and self management interventions [6]. To guarantee longitudinal continuity, chronic patient outcomes are measured systematically through structured health tracking instruments and recorded in the patient’s medical record, in order to prevent a relapse into poor health condition after improvement. The caregiving team also promotes the creation of networks for vertical continuity, sharing clinical information with other providers serving the same population (e.g. hospitals or private practices). As, in the most recent community models, members of collaborative teams share the same centralized building (primary care centres), this setting can also benefit from diagnostic and treatment technologies for early disease detection and rehabilitation that could avoid non-urgent access to accident and emergency department (A&ED) and considerably reduce the number of unnecessary referrals to specialists.

Despite the apparent superiority of team-based community models, these solutions have shown some important limitations, as highlighted in the present trial as well as in the national and international literature. According to Lamarche et al. [5], although on the whole such models (integrated community models in the author’s taxonomy) achieve the best empirical results in terms of effectiveness, cost reduction, care continuity, quality and equity, they encounter difficulties in preserving the individual relationship between the patient and the professional mostly responsible for their care (relational continuity). This situation generates poorer responsiveness and limits access. Besides, the findings of an empirical study using administrative data showed a considerable variation in medical patterns among some Italian primary care providers organised on a team-based model [7].

Thus, while it is possible to recognise both strong and weak points in the various primary care models, it is still difficult to determine which solution is the best, especially in terms of patient preference. To design services that are sensitive to population needs in a context of limited resources it is therefore important to find out which aspects of primary care models users/patients would most like to see improved, given that they cannot have the best level of every characteristic. This implies a necessary trade-off between the most important attributes of the aforementioned models from the population perspective.

At present, although several studies have elicited preferences for various aspects of the primary care sector, there is scant evidence on the importance of different primary care models. Many studies have focussed mainly on family practice, investigating patient predilections [8–10] and patient and provider preferences [11] for characteristics connected with a GP appointment—mainly access and type of professional consulted. The value given by the population to continuity of care [12] and regarding the provision of nurse-led versus doctor-led primary health care was also taken into account [13]. Concerning out-of-hours services, preferences for general [14, 15] and paediatric [16] out-of-hours primary care services were quantified, as well as the importance of attributes associated to emergency primary care services available during GP hours [17]. Other studies evaluated the importance of different aspects of the doctor–patient relationship in general practice [18–22]. Regarding publications investigating provider choices, GPs’ preferences for different job characteristics in general practice have been elicited [23–26] and community pharmacists’ priorities for existing and potential new roles in primary care have been examined [27]. Recently a Swedish study [28] reported population preferences for alternative primary care settings, which, however, considered a specific primary care system, different from the Italian one, and did not take into account factors related to respondents’ experience.

In this study, a discrete choice experiment (DCE) was used to elicit patient preferences for different primary care models. DCEs are a popular stated preference technique in health economics [29] that elicit people’s preferences on the basis of their stated preferences in hypothetical scenarios or choice sets [30].

Through the use of a DCE, the relative importance of the different attributes for a sample of Tuscan (Italian) residents and for respondent subgroups was examined, as well as the rate at which individuals trade between attributes. The relative value of different primary care service configurations was also investigated.

## Methods

### Measurement of preferences

In the 2008–2010 regional health plan [31], the Tuscan regional health system (TRHS) introduced the strategic priority of developing a proactive approach to population-based medicine, experimenting with inter-professional team-based arrangements focussed specifically on chronic patients (“primary care units”). Within this context, a DCE was embedded in the 2009 patient satisfaction and experience survey on primary care services (SEPC) which was developed by the Laboratorio Management and Sanità of Scuola Superiore Sant’Anna on behalf of Tuscany Region [32].

The SEPC questionnaire was administered to a sample of Tuscan residents over 18 years of age and consisted of four sections. The first presented questions taken from the SEPC survey about respondents’ experience with primary care services, such as the frequency of GP visits in the last year, the reason for seeing the GP, whether the patient had or had not been listened to carefully by the GP, and whether the GP had given clear explanations about the treatment or not. In the second section, the attributes of primary care services selected for the DCE experiment were presented, after a short introduction on why the DCE was performed. To identify participants that appear unwilling to trade-off the attributes, each respondent was invited to rank the attributes in order of importance. In the third section, participants were asked to make their choices in the context of a consultation for a non-urgent problem, and to express their preference for each choice set presented by selecting one of the unlabelled options A or B, considering that all other characteristics about the consultation were assumed to be equal. This section started with an exhaustive description of each attribute and of its level to clarify their meanings and implications. The last section consisted of questions on current health status as well as socio-demographic questions, taken from the SEPC survey.

Although DCEs in health care have been carried out mainly using self-completed postal questionnaires [29, 33], a computer-aided telephone interview approach was selected as it allowed a wide geographic coverage with higher response rates than postal or internet approaches [34] and it was considered a viable method if used with a small number of choice sets per respondent [35].

The sample was stratified into the 34 health districts in the region. In each health district, a sample size of approximately 196 subjects was required, assuming that a proportion of 50 % of the adult population have used primary care services in the last 12 months at a 95 % confidence level with a margin of error of ±7 %. Assuming a response rate of approximately 40 %, which is in line with previous studies in that area, oversampling was performed to ensure that the minimum sample size was obtained. The calculated sample size was then multiplied by 34 to obtain the total sample size of 6,970. However, there is limited guidance on sample size calculations for DCEs, and there are no practical well-designed rules to guide the analyst [36]. Pearmain et al. [37] have suggested that, for DCE designs, sample sizes over 100 are able to provide a basis for modelling preference data, and Hensher et al. [36] have suggested a rule of thumb of 50 respondents per question to provide adequate variation in the variables of interest. Telephone interviews were conducted in the spring of 2009 by a team of 13 experienced interviewers. To minimise interviewer effects, interviewers were initially trained before taking part in the study.

### Selection of attributes, levels, and scenarios

Through a review of the existing literature and semi-structured interviews to primary care managers and managers of LHAs, attributes and levels describing the different primary care scenarios in the choice experiment were identified. These were than validated in a focus group. The number of selected attributes regarding waiting times, kind of primary care provider and presence of diagnostic facilities, was limited to the three most important factors that emerged [38, 39], in order to avoid placing a significant cognitive burden on respondents [33]. Plausible levels were assigned to each of the attributes (Table 1), taking into account also the results of previous choice experiments [15, 28].

**Table 1:** Discrete choice experiment (DCE) attributes, levels and names

Attributes	Level	Name
Waiting time for visit (WAIT)	0 min	Waiting time
	90 min	
	180 min	
Primary care provider (GP)	One’s own GP	Own GP
	A primary care team (GP + other professionals)	Primary care team
	Another GP in the same practice^a^	Another GP
Diagnostic facilities (DIAG)	A lot of diagnostic facilities	Many diagnostic facilities
	Some diagnostic facilities	Some diagnostic facilities
	A few diagnostic facilities^a^	Few diagnostic facilities

*GP* General practitioner

^a^Denotes the base category

### Experimental design and construction of choice sets

A full factorial design with 3^3^ (27) combinations was used. To obtain a more statistically efficient design, the 27 alternatives were paired into choice sets using systematic level changes [40]. This approach preserves orthogonality, level balance and minimal overlap [41]. The exclusion of an opt-out option could be a violation of the underlying welfare measures of the economic experiment, since it makes it impossible to estimate the value of doing nothing, which may be chosen in practice [42]. Nevertheless, this may raise the number of neutral responses, increasing the number of individuals that may choose the opt-out scenario to prevent making difficult choices, even though this would not provide the highest utility [43]. The feasibility of using an opt-out approach—adding a “neither” option—was tested in the pilot study, that revealed that neutral responses were likely to be obtained in this DCE; therefore a forced choice was chosen as appropriate. Adding a status quo alternative would have been another option, but it was rejected for two reasons. First of all, the “status quo bias”, i.e. the tendency to choose what respondents know best [44], since respondents were already experienced with primary care services. Secondly, there were possible econometric and interpretation difficulties, due to the fact that the status quo alternative differed among respondents.

A few alternatives to come out of the design may not contain feasible attribute-level combinations (specifically “own GP” associated with “many diagnostic facilities”). Since the pilot testing indicated that individuals did not find any of the combinations in the experimental design implausible, constraints between levels were not applied [45].

A pre-pilot test was performed to a sample of 34 individuals of different ages and from different geographical locations (health districts).

Considering that there is little evidence in the literature about the manageable number of choice sets per respondent with telephone surveys and that, above all, the appropriate number of choice sets is context-specific [29], a blocked design was used to pre-pilot two different sets of questionnaires, including ten and four choice tasks, respectively. The 27 choice sets were therefore distributed across three blocks of nine and nine blocks of three, respectively, creating an extra column with a number of levels equal to the number of blocks, which is uncorrelated with every attribute of every alternative. Level balance was satisfied within each block. In each version, the sequence of questions was randomised and the first choice set was then repeated as the last choice set, to provide a check of response consistency (discussed further below) and to allow for a “warm-up” question at the beginning of the sequence [46]. As it adds no statistical information, the repeated question was not included in the main data analyses. At the end of the choice experiment, respondents were asked if they were taking into consideration other attributes not included in the task when making choices, and to outline them in the affirmative case.

On the basis of respondents’ direct feedback, response rates, item response rates, and rationality tests, the pre-piloting indicated that respondents were able to handle a maximum of four choices. Apart from the “consultation length” mentioned by one respondent, no other attributes different from those included in the DCE were considered as relevant by the participants during their decision making process. Some changes were made to the wording of the questions and the instructions, integrating in particular the attributes description with examples in order to place the hypothetical scenarios in a more recognisable and realistic setting.

A further pilot study was undertaken with a new sample of 34 subjects of different age and geographical locations. On the whole, respondents understood the choice tasks, finding the questionnaire acceptable.

**Fig. 1:**
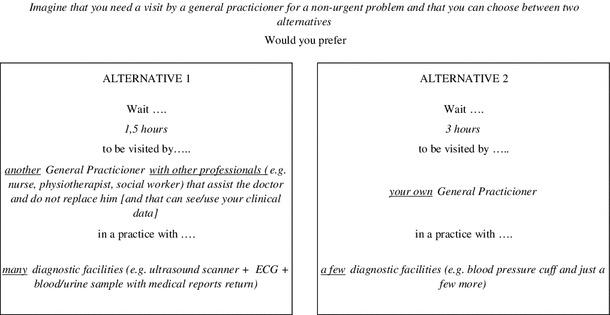
Example of a choice set

Thus, in the final questionnaire, each respondent was assigned randomly to one of the nine blocks and was presented with four discrete choices. To preserve data set orthogonality [47], the nine subgroups related to each questionnaire version included an equal number of respondents. The groups were then tested for homogeneity with regard to geographical location, age and sex. An example of a choice task is shown in Fig. 1.

The preliminary detection of “dominant options” (where all attributes of the first alternative are preferred to all attributes of the second alternative, or vice versa [48]) was not feasible in this DCE for two reasons. First, the experiment includes a qualitative attribute (“primary care provider”) with levels that do not have a clear ordering and that vary systematically across the alternatives. Second, the sample size of the pilot study was inadequate to make reliable prior assumptions on parameters. Nevertheless, potential imprecision in the estimates should predictably be filtered out, since design techniques that also account for statistical efficiency, excluding most of the choice situations with clearly dominant options [49], were used, and also because of the large sample size of the study.

### Estimation procedure

Choice data were modelled using a random utility maximisation framework [50]. Each participants’ choice between pairs, treated as a single observation, was included in the model as the binary dependent variable (“1” represents the option being chosen, while “0” not chosen). The independent variables were the differences between the levels of each attribute in each pair of scenarios. A random effect probit model was used for the estimation [51], to represent the distribution of the error term that was assumed normal, and also to account for multiple observations from a single respondent. Having also assumed a linear additive utility function, the follow baseline empirical model was specified:

1 $$\varDelta U_{nc} = \beta_{0} + \beta_{1} *\varDelta {\text{WAIT}}_{c} + \beta_{2} *\varDelta {\text{GP}}_{c} + \beta_{3} *\varDelta {\text{DIAG}}_{c} + \pi_{n} + \lambda_{nc}$$

Δ*U* indicates the difference in utility between alternatives of a choice set that is observed indirectly. The subscripts *n* and *c* refer to the individual and the number of choice set, respectively. ΔWAIT, ΔGP, ΔDIAG represent the differences in attribute levels within each choice set. In view of the fact that a shorter waiting time and more diagnostic facilities are intuitively preferable, it was expected that the former attribute would be associated with a negative coefficient and the latter with a positive one. For the remaining qualitative attribute, no a priori assumption was made [52]. *β*_0_ is the constant term, included to test and control model misspecifications due to unobserved dimensions or unobserved interactions between respondents’ socio-economic characteristics and dimensions [23]. *β*_1_, *β*_2_, *β*_3_ denote the part-worths estimated from the regression analysis. *π*_*n*_ is the individual specific error term whereas *λ*_*nc*_ is the random error term [53]. To quantify the correlation between choices, the serial correlation was estimated, or Corr [*π*_*ν*_, λ_*νχ*_] = *ρ*. Effects-coding [54] was used for the attributes “primary care provider” and “diagnostic facilities” (−1 for the base category, 1 for the presence of another category and 0 otherwise). It was also hypothesised that respondents’ socio-demographic condition and their past experience with the GP would influence preferences for a primary care service. These characteristics were entered into the model analysis through interactions with the main effects. The segmented model, which included all main and interaction effects, was reduced stepwise to a more parsimonious one, by excluding insignificant interaction effects one at a time on the basis of the likelihood ratio test with a *P* value >0.05 [55].

The estimated marginal utilities were used to quantify the relative importance of the attributes and the marginal rate of substitution (MRS), calculated by dividing the estimated values of the attributes with the value of the “waiting time” attribute [56]. For each effects-coded variable the marginal utilities were obtained calculating the difference between the estimated coefficients and the base category coefficient, defined as the negative sum of the coefficients for all other categories of that variable [26].

Furthermore, as previously done in other health care related DCEs [15, 57–60], the part-worth utilities (*β*s) and the constant estimated in the Eq. 1 were summed to predict the overall utility for all the combinations of attribute levels in the full factorial design. In addition to the 27 hypothetical scenarios included in the design, three additional forms of care delivery, which are the most representative of the Italian primary care service alternatives previously described (“solo general practice”, “group general practice” and “primary care centre”), were identified. For a visit to a “solo general practice”, on average, patients have to wait more than 1 h (70 min) to be seen by their own GP exclusively, in a practice with few diagnostic facilities. In a “group general practice”—often a setting with some diagnostic services—if patients accept to be seen by an associated GP different from their own physician, they usually have to wait less time (40 min). A consultation in a “primary care centre” normally implies a short waiting time (10 min), for a visit provided by a primary care team (the GP and other professionals) in a practice with many diagnostic services. All the resulting scores were then ranked in order of preference.

The 95 % confidence intervals (95 % CIs) for the “willingness to wait” estimates and predicted utilities were calculated using non-parametric bootstrapping [61] with 2,000 iterations. All statistical analyses were performed using Stata 10 (StataCorp, College Station, TX).

### Tests of the validity of responses

Internal validity was tested by three approaches: (1) consistency of preferences, (2) willingness to trade, and (3) consistency with theoretical predictions.

1. To measure internal consistency, a test of stability was carried out, by which subjects are asked to consider the same discrete-choice comparison both at the beginning and at the end of the questionnaire. We expected subjects to make the same choice both times the question was offered.
2. The willingness of respondents to trade-off the attributes was tested through the approach used in Scott et al. [62], identifying respondents with dominant preferences (individuals that always choose according to the best level of a given attribute). In relation to the attributes “waiting time” and “diagnostic capabilities”, where the “best” could be identified, for each attribute was tested whether an individual always chose the option with the best level and ranked the attribute as the most important in a simple ranking of the attributes. Dominant preferences for “primary care provider” were not calculated since the “best” level of this qualitative attribute was not known a priori. The influence of dominant preferences was then assessed by running a regression analysis twice, including and excluding respondents with dominant preferences.
3. Theoretical validity was investigated by examining the sign and significance of parameter estimates.

## Results

Of the 6,970 persons contacted, 3,372 participated in the SPEC survey. Of these participants, 3,263 completed the choice tasks, with a response rate of 47 %.

The respondents were distributed equally and without any significant differences in socio-demographic characteristics and past experience with the GP across the nine versions of the questionnaire used. Details on responders’ characteristics are given in Table 2.

**Table 2:** Characteristics of respondents

Attribute	Level	Name	Frequency	%
Age group	18–49 years	Age 18–49	955	29.6
	50–69 years	Age 50–69	1,388	43.0
	>69 years^a^	Age >69	886	27.4
Gender	Female	Female	2,502	76.8
	Male^a^	Male	756	23.2
Education	None/primary level	Educ no	1,047	32.6
	Secondary level	Educ sec	1,833	57.1
	University degree or higher^a^	Educ uni	328	10.2
Employment status	Not working/retired	Empl no	1,490	46.5
	Working (high-skilled jobs)	Empl high	308	9.6
	Working (medium/low-skilled jobs) + students^a^	Empl low	1,406	43.9
Income	High	Inc high	1,313	42.2
	Medium	Inc med	1,279	41.1
	Low^a^	Inc low	520	16.7
Living alone	Yes	Alone	402	12.5
	No^a^	Alone no	2,802	87.5
Health status	Fair/poor	Health low	420	13.1
	Excellent/very good/good^a^	Health high	2,775	86.9
Chronic disease	Yes	Chron	1,254	38.9
	No^a^	Chron no	1,971	61.1
Frequency of visits to GP clinic in the last year	Never/from 1 to 3 times	Freq low	921	31.1
	More than 3 times^a^	Freq high	2,043	68.9
Reason for seeing the GP	General health check/minor illness treatment	Reas min	504	17.3
	Already existing illness check	Reas exist	666	22.8
	Prescriptions/certificates/other^a^	Reas other	1,748	59.9
The GP works with other GPs	Yes	Assoc	839	28.8
	No^a^	Assoc no	2,079	71.2
Time you waited in the clinic	<1 h	Wait less	2,180	77.1
	>1 h^a^	Wait more	648	22.9
You have had to put off seeing the GP	Yes (waited too much, GP unavailable, clinic closed)	Putoff	285	9.8
	No^a^	Putoff no	2,633	90.2
The GP listened to you carefully	Yes	Listen	2,870	98.6
	No^a^	Listen no	42	1.4
The GP gave you enough time to discuss	Yes	Entime	2,870	98.5
	No^a^	Entime no	43	1.5
The GP involved you in the decisions	Yes	Involv	2,839	97.7
	No^a^	Involv no	68	2.3
The GP gave you clear explanations	Yes	Clear	2,871	98.7
	No^a^	Clear no	38	1.3
The GP gave you advices	Yes	Advice	1,965	67.4
	No^a^	Advice no	949	32.6
You trust in your GP	Yes	Trust	2,878	98.6
	No^a^	Trust no	40	1.4

^a^Denotes the base category

### Sample characteristics

The age of respondents ranged from 18 to 96 years old with a mean age of 58 years; 76.8 % were female and 57.1 % had a secondary level of education. 43.9 % were working in medium and low skilled jobs or engaged in a full time education; 41.1 % had a medium income. 13.1 % were in a fair or poor health status and 38.9 % of them declared a chronic disease. 68.9 % of respondents had visited their GP clinic more than three times in the last year and 77.1 % of them waited <1 h for a consultation. The GP was seen mainly in order to get a prescription or certificate (59.9 %) and to check on a pre-existing illness (22.8 %). During the consultation, the GP listened carefully to 98.6 % of participants, gave 98.5 % of them enough time to discuss their problem, involved 97.7 % of them in decisions, gave clear explanations to 98.7 % of them and advice on eating or physical activity to 67.4 % of them. 98.6 % of respondents trusted their GP.

### Internal validity

A total of 11 % of respondents failed the stability test, which was considered to be acceptable as the percentage of inconsistent responses usually varies from 1 % [63] to 25 % [57].

Also, 11 % of respondents always chose the scenario with the best level of a given attribute that they ranked as the most important. The level of dominant preferences was similar to other studies [62]. The results of regression analyses indicated that the impact on the coefficient size and direction for each attribute was the same regardless of whether dominant preferences were excluded or included within the data analysis. Considering also that random utility models are robust to violations of compensatory decision making [64], all respondents were thus included in the final analysis.

### Main effects model

The serial correlation obtained from running the random effect models was close to zero and not statistically significant, indicating that respondents treated the decision made in each pair-wise comparison as a separate hypothetical situation and not in association with the choice made in each of the remaining pair-wise comparisons. Hence, all models were re-fitted to the data using the standard probit model.

To verify whether the linear representation of the continuous variable “waiting time” was admissible, a univariate smoothed scatter plot [65] was first performed to show potential non-linearities. In addition, the probit model was re-estimated using dummy variables replacing the continuous variable. Lastly, a likelihood ratio test was used to assess whether the inclusion of a quadratic term would have improved the explanatory power of the model. The results confirmed that a linear representation was congruent with the data.

As Table 3 shows, the main effects probit model has a good fit (McFadden Pseudo *R* ^2^ = 0.25) and predicts correctly 76 % of the responses. All the attributes had a significant impact on respondents’ decisions.

**Table 3:** Regression results from DCE: main effects model

Attribute	Coefficient	SE	MRS (min)	95 % CI (lower)	95 % CI (upper)
Constant	0.546***	0.016	–	–	–
Waiting time (min)	−0.006***	0.000	–	–	–
Own GP	0.611***	0.015	207.1	196.5	219.5
Primary care team	0.100***	0.014	127.1	118.1	135.7
Many diagnostic facilities	0.534***	0.015	194.9	184.9	205.7
Some diagnostic facilities	0.176***	0.014	138.8	129.6	148.4
*N*	19,340				
Log likelihood	−10,086.97				
Likelihood ratio test (*c* ^2^, df)^a^	6,636.99 (5)***				
Pseudo *R* ^2^ McFadden^a^	0.248				

*SE* Standard error,* MRS* marginal rate of substitution

*** *P* < 0.001

^a^Compared to a only constant model

Other things being equal, participants would be willing to wait up to 207 min for a consultation with their own GP, 195 min longer to be visited in a setting with many diagnostic services and 139 min longer to have some diagnostic facilities in the practice. Therefore, patients preferred to be visited by their own GP and a practice with many diagnostic facilities to a practice with some diagnostic services. These results are in line with expectations and provide support for the theoretical validity of the model.

### Segmented model

Compared with the main effects model, the reduced model has a better fit (Pseudo *R* ^2^ = 0.28), with the main effects of a similar sign and significant. The results of the model are given in Table 4.

**Table 4:** Regression results from DCE: segmented model

Attribute	Coefficient	SE
Constant	0.5993***	0.018
Waiting time	−0.0065***	0.000
Own GP	0.6165***	0.020
Primary care team	0.1636***	0.019
Many diagnostic facilities	0.5507***	0.019
Some diagnostic facilities	0.2563***	0.028
Waiting time × age 18–49	−0.0006***	0.000
Waiting time × health low	0.0003*	0.000
Waiting time × wait less	−0.0004**	0.000
Own GP × empl no	0.0740***	0.016
Own GP × chron	0.3579***	0.018
Own GP × freq low	−0.0676***	0.018
Own GP × advice	0.1159***	0.017
Primary care team × age 18–49	0.0927***	0.023
Primary care team × age 50–69	−0.0591**	0.019
Primary care team × chron	−0.2379***	0.017
Primary care team × freq low	0.0606**	0.018
Primary care team × advice	−0.0919***	0.017
A lot of diag. facilities × female	0.0360*	0.017
A lot of diag. facilities × chron	0.1065***	0.015
Some diag. facilities × age 18–49	0.1166***	0.026
Some diag. facilities × age 50–69	−0.0407*	0.019
Some diag. facilities × empl no	0.1091***	0.027
Some diag. facilities × empl high	−0.1024**	0.033
Some diag. facilities × reas min	0.0481**	0.018
Some diag. facilities × putoff	0.0829***	0.024
*N*	16,120	
Log likelihood	−8,055.42	
Likelihood ratio test (*c* ^2^, df)^a^	6,236.23 (25)***	
Pseudo *R* ^2^ McFadden^a^	0.279	

*** *P* < 0.001, ** 0.01 > *P* ≥ 0.001, * 0.05 ≥ *P* ≥ 0.01

^a^Compared to a only constant model

The marginal utilities indicate that younger respondents (under 45 years) and people that waited in the GP practice <1 h for the last visit would prefer short waiting times. Chronic patients and those who are retired or are not working preferred to be visited by their own GP. This provider had also a higher marginal utility for respondents who, in their last consultation, received advice from the GP on eating or physical activity. On the other hand, the primary care team was the favourite option for younger respondents who went less frequently to the GP clinic in the last year.

Concerning the impact of respondent characteristics on preferences for the diagnostic setting, a visit in a practice with many diagnostic facilities was the preferred option for female respondents and for those that reported a chronic condition. A setting with some diagnostic services was more likely to be preferred by younger respondents and by people who are retired or are not working. Those who saw a GP in the last year for treatment of a minor illness or for a general health check and those who have had to put off seeing the GP in the last year also preferred to be visited in a practice with some diagnostic facilities.

### Predicted utilities

With regards to the choices between the different service configuration, Table 5 presents a utility-based ranking for all the configuration hypothesized.

**Table 5:** Predicted utilities for alternative scenarios of care delivery

Scenario	Waiting time	Caregiver	Diagnostic facilities	Indirect utility^a^	95 % CI (lower)	95 % CI (upper)
22	0 min	Own GP	Many	3.11	3.03	3.19
25	0 min	Own GP	Some	2.75	2.68	2.83
18	0 min	Primary care team	Many	2.60	2.52	2.68
Primary care centre	10 *min*	Primary care team	Many	*2.54*	*2.46*	*2.61*
13	90 min	Own GP	Many	2.54	2.46	2.61
6	0 min	Primary care team	Some	2.24	2.17	2.32
7	90 min	Own GP	Some	2.18	2.11	2.25
26	90 min	Primary care team	Many	2.03	1.95	2.10
2	180 min	Own GP	Many	1.96	1.88	2.05
20	0 min	Own GP	Few	1.87	1.82	1.92
10	0 min	Another GP	Many	1.79	1.74	1.84
12	90 min	Primary care team	Some	1.67	1.59	1.75
16	180 min	Own GP	Some	1.60	1.52	1.69
9	180 min	Primary care team	Many	1.45	1.36	1.53
14	0 min	Another GP	Some	1.43	1.38	1.48
Solo GP	*70* *min*	Own GP	Few	1.42	1.37	1.47
1	0 min	Primary care team	Few	1.36	1.31	1.40
5	90 min	Own GP	Few	1.29	1.24	1.35
19	90 min	Another GP	Many	1.22	1.16	1.27
Group GP	*40* *min*	Another GP	Some	1.18	1.13	1.23
21	180 min	Primary care team	Some	1.09	1.01	1.18
3	90 min	Another GP	Some	0.86	0.80	0.91
23	90 min	Primary care team	Few	0.78	0.73	0.83
11	180 min	Own GP	Few	0.72	0.65	0.78
4	180 min	Another GP	Many	0.64	0.58	0.71
8	0 min	Another GP	Few	0.55	0.55	0.55
24	180 min	Another GP	Some	0.28	0.22	0.35
15	180 min	Primary care team	Few	0.21	0.14	0.27
17	90 min	Another GP	Few	−0.03	−0.05	0.00
27	180 min	Another GP	Few	−0.60	−0.66	−0.55

^a^Indirect utility = 0.546 + [−0.006 × waiting time (min)] + (1.321 × Own GP) + (0.811 × primary care team) + (1.243 × Many diag. facilities) + (0.885 × Some diag. facilities)

Among the existing care models, the “primary care centre” would be the most preferred scenario, followed by the “solo general practice” and the “group general practice”.

Despite “own GP” being the most preferred respondents’ caregiver, the actual context in which such physicians have to operate (“solo general practice”)—with few diagnostic facilities and long waiting times for the visits—would not be considered the best service alternative.

With reference to the “primary care centre”, most of all, its superiority over the “solo general practice” derived from a grater diagnostic potential, rather than a shorter waiting time. Indeed, assuming that the former service would not maintain a certain diagnostic power, even with no waiting time for the visit (scenario 1) it would have a lower benefit score compared with the utility of the “solo general practice”.

Regarding the “group general practice”, to have a higher benefit score with such service configuration compared with the utility of the “solo general practice”, respondents need to be compensated with a 40-min reduction in waiting time (scenario 14) or, assuming no changes in waiting time, with more diagnostic services.

## Discussion and conclusion

The results presented in this paper provide useful insights regarding community preferences for different primary care models. To the authors’ knowledge, this is the first large-scale study in this context that takes into account the impact of the diagnostic facilities.

The “willingness to wait” values have shown that a consultation with one’s own GP is more important than being seen by a primary care team and than a practice with many diagnostic services. This highlights an important finding given the tendency to limit relational continuity in current health policies [66]. The respondents’ predilection for their own GP was also highlighted in similar studies [8, 10, 22].

Preferences differed also by respondents’ characteristics and past experiences, and some of the interaction effects that emerged were similar to those described by previous authors. Consistently with the interactions found we can suppose that people who preferred to be visited by their own GP and who did not choose to be visited by a primary care team might be those with high and continuous healthcare needs, probably living in a certain degree of isolation, which makes it difficult to seek new or alternative care providers. Such group could see their own GP as a stable reference point. On the other hand, respondents who preferred a consultation with a primary care team regardless of their own GP are young people, with low healthcare needs, that probably have not yet developed a type of dependency on their own GP, and could therefore be more sensitive to service innovations. Regarding subgroup preferences for diagnostic technologies, we can presume that people who preferred to have many diagnostic facilities in the practice are probably those with a greater willingness to pay and with high healthcare needs. On the other hand, individuals who had a preference for some diagnostic technologies and who did not choose a practice with many diagnostic facilities seem to be those with a lower willingness to pay, with minor healthcare needs and without a propensity to wait too long for a diagnostic test.

The results obtained from the predicted utilities of different service configurations would need to be combined with the costs of different combinations of attributes to establish the most cost-effective model of care. Nevertheless, these results have important implications for the demand for new primary care models. Considering that the Primary Care Centre would perform better than the “solo GP” even with some diagnostic services (for example scenario 6), for a more rapid diffusion of this model, policymakers and managers, at least in the first phase, may direct care provided by Primary Care Centres towards a younger population with low healthcare needs. This group, indeed, has demonstrated a strong preference for this specific service configuration. This strategy, of course, would only partially consolidate team-based community models, because such organisations were particularly designed to tackle the needs of the chronic sick and of elderly people. However, it could be considered a valid alternative to the inappropriate use of A&ED services (non urgent access).

Future policies to improve primary care organisations should be based on a broader framework that takes into account the different needs of population sub-groups, balancing responsiveness with care continuity, equity, and appropriateness. In this context, the present study, following on from previous research [67], provides evidence on how the Tuscan healthcare system is moving toward a pro-active approach in order to provide differentiated services specific to different healthcare needs.

This study, however, has some limitations that should be considered. First of all, the relatively small number of attributes used in the study and the exclusion of factors that appeared as relevant in other studies, such as “consultation length” [11, 24], in order to avoid placing a considerable cognitive burden on respondents, may have led to the omission of other characteristics that may have been captured within the constant term. There is little discussion on this in the literature, and where a significant constant is identified, the problem tends to be ignored [68]. While attempts were made to select the attributes in an unbiased way, it is not possible to establish whether other qualitative approaches would generate the same attributes and levels. For a better evaluation of the significance of the attributes found, comparative qualitative approaches identifying attributes and levels for the same study would be necessary [25]. Secondly, even though the response rate achieved was in line with other DCEs in similar settings [14, 16, 18, 19], a comparison with Tuscan population data revealed that older respondents and women were overrepresented. This result may be expected in an “in-home” interview survey of this type [69]. A more ample evaluation of sample selection bias was not feasible because age and sex were the only available data on the Tuscan population. The potential for such biases needs to be addressed in future studies, by allocation of more resources at the recruitment phase. Thirdly, potential perceptions of implausible attribute-level combinations could have reduced response efficiency, deteriorating the precision and accuracy of parameter estimates [70]. Although the pilot revealed that individuals did not find the alternatives in the choice tasks unrealistic, identification of this potential bias was not practical given the lack of data on the actual primary care services received by respondents [29]. Future DCEs should attempt to follow up model estimation with focus groups to address the validity of the results achieved by asking respondents if their preferences are consistent with the findings of the estimated model. Furthermore, collecting information on the respondent’s current primary care service would also prevent the adoption of a forced choice design, for instance, using such information for the construction of a status quo alternative in the choice set. Fourthly, the rich set of respondents’ characteristics, incorporated into the model through the interaction with the attributes, allowed us to show several aspects of preference heterogeneity. However, some differences in tastes will probably remain random to the extent that it cannot be related to observed characteristics. Future analyses should explore the added value of discrete choice models that relax the assumption of taste homogeneity (e.g. mixed logit and latent class model) by allowing for random taste variation.
